# The role of ultrasound and mitofusin-2 levels to predict pregnancy outcomes in patients with severe preeclampsia: a case-control study

**DOI:** 10.1590/1806-9282.20240152

**Published:** 2024-08-16

**Authors:** Kazım Uçkan, Çağdaş Özgökçe, Yusuf Başkiran, Ömer Gökhan Eyisoy, İzzet Çeleğen, Halil İbrahim Akbay

**Affiliations:** 1 Yuzuncu Yil University, Faculty of Medicine, Gynecology and Obstetrics Clinic - Van, Turkey.; 2 Zeynep Kamil Women and Children Research Hospital - İstanbul, Turkey.; 3 Yuzuncu Yil University, Faculty of Medicine, Department of Public Health - Van, Turkey.

**Keywords:** MFN2 protein, Preeclampsia, Umbilical artery, Uterine artery, Arterial pressure

## Abstract

**OBJECTIVE:**

The aim of this study was to evaluate mitofusin-2 levels and fetal Doppler ultrasonography effects in patients with severe preeclampsia.

**METHODS:**

This single-center case-control study was conducted in the gynecology service of the university hospital in Van. A total of 90 pregnant women aged 18-40 years were included in the study. Of these, 30 are normal, 30 have mild preeclampsia, and 30 are pregnant with severe preeclampsia. In this study, especially in severe preeclampsia patients, serum mitofusin-2 levels and important fetal Doppler flows such as uterine arterial pressure, umbilical arterial pressure, and 1st and 5th minute Apgar scores, birth weight, and the relationship between postnatal outcomes such as week of birth and the number of patients in the neonatal intensive care unit were investigated.

**RESULTS:**

There was a significant difference between the three groups in terms of mitofusin-2 levels, which was the highest in the group (p<0.05). Maternal serum mitofusin-2 levels were positively correlated with uterine arterial pressure (r=0.543, p=0.007), umbilical arterial pressure (r=0.238, p=0.008), diastolic blood pressure, and systolic blood pressure (p<0.001). Receiver operating characteristic curve of mitofusin-2 in predicting preeclampsia is as follows: optimal cutoff 1.6 ng/mL; area under the curve: 0.861; 95%CI: 0.786-0.917; sensitivity: 83.9%; and specificity: 70.0%, (p≤0.001). A one-unit increase in mitofusin-2 resulted in a statistically significant 4.21-fold increase in preeclampsia risk.

**CONCLUSION:**

This study recommends the use of mitofusin-2 together with fetal Doppler ultrasound findings as a reliable indicator of preeclampsia severity.

## INTRODUCTION

Preeclampsia is a disease that manifests itself with hypertension and proteinuria that occurs after the 20th gestational week and can affect many organs. It is one of the important reasons for neonatal and maternal morbidity and mortality. PE can affect 2-8% of pregnant women worldwide^1^. Many factors such as hypoxia, endothelial dysfunction, mitochondrial dysfunction, inflammation, and oxidative stress have been listed among the causes of PE, the etiology of which has not been fully explained^2^.

Although not fully understood, the pathophysiology of preeclampsia is likely a multifactorial condition consisting of genetic and environmental factors and abnormal placentation. Current evidence shows that preeclampsia is a two-stage disease. The first stage is the asymptomatic stage of early pregnancy resulting from poor placentation due to abnormal trophoblast invasion and spiral artery remodeling. This results in the second stage of the disease, characterized by placental ischemia/reperfusion injury and maternal immune-mediated response. As a result, there is a release of anti-angiogenic factors and placental debris into the maternal circulation and an inadequate release of pro-angiogenic factors. This leads to angiogenic imbalance, immune-mediated exaggerated inflammatory response, and endothelial cell dysfunction, resulting in increased platelet aggregation, abnormal activation of the coagulation system, and systemic vascular augmentation. The general outcome of this stage is clinical symptoms such as high blood pressure, proteinuria, and other end-organ damage^3^.

Poor placentation results in abnormal fetal perfusion. This condition is associated with abnormal uterine artery blood flow and an increased incidence of fetal growth restriction in pregnancies affected by preeclampsia, especially in preterm pregnancies. This abnormal perfusion often appears as notching on uterine artery Doppler evaluation. However, the usefulness of this finding in predicting preeclampsia is limited^4^.

Studies have shown that increased placental hypoxia causes mitochondrial dysfunction and is associated with PE. In addition, it has been determined that there is placental mitochondrial dysfunction in patients with PE and there is a difference in the amount of placental mitochondrial protein^5^. Reactive oxygen species (ROS) occur due to ischemia and hypoxia following inappropriate trophoblastic invasion and placentation. With their participation in the maternal circulation, an increase in the systemic inflammatory response occurs^6^.

Mitochondria is a structure in which morphological changes occur through processes called fission (dividing into smaller parts) and fusion (joining parts). There is a mitochondrial fusion protein 2 (Mfn-2) in the outer membrane surrounding the mitochondria inside the cell. Mfn-2 is a protein involved in the regulation of many cellular processes (fusion, morphology and function, energy metabolism, signal transduction, proliferation, and apoptosis)^7^.

This is the first study to investigate the effect of maternal serum Mfn-2 levels in patients with sPE.

## METHODS

### Ethical aspects

Ethics committee approval was obtained for this prospective study (approval number 2022/08-01). The researchers committed to complying with the Declaration of Helsinki guidelines for medical research in humans throughout the study (approval number: 2022/08-01).

### Study design

This single-center case-control study was conducted in the gynecology ward of the university hospital in Van. No sample was selected for the study. The study was conducted with a total of 90 patients, aged between 18 and 40 years, including 30 with mild preeclampsia (mPE), 30 with sPE, and 30 normal patients, who met the determined criteria. Mild and severe preeclampsia (sPE) were diagnosed according to the American College of Obstetricians and Gynecologists (ACOG) guidelines^8^. The control group consisted of normotensive pregnant women who applied to our clinic in the same period, had a single pregnancy, and were compatible with the maternal age and gestational age. To avoid selection bias in the study, the case group was determined according to the preeclampsia classification criteria in the ACOG guide. The control group was randomly selected from pregnant women with similar age, gravida, parity, gestational week, miscarriage history, and body mass index (BMI) to the case group.

### Data collection tools

Two PE groups were formed: mild and severe. Pregnant women with blood pressure ≥140/90 mmHg, random proteinuria (+), or urine protein ≥300 mg/24 h measured every 4 h after the 20th week of pregnancy were included in the mPE group. Diastolic blood pressure (DBP) >110 mmHg or systolic blood pressure (SBP) >160 mmHg in two separate measurements taken at least 4 h apart, neurological (headache, visual disturbances, or seizures), hepatic (epigastric pain, pain in the right hypochondrium, transaminase more than twice the upper limit of normal), renal pregnant women with hematological (thrombocytopenia, hemolysis), or acute pulmonary edema (serum creatinine elevated >1.1 mg/dL or more than twofold increase in baseline creatinine) were included in the severe preeclampsia group. BMI was obtained by dividing weight (in kg) by the square of height (in m).

The following variables were collected for both groups: age, gravida, parity, history of abortion, mode of delivery (vaginal or cesarean section), birth weight, week of birth, neonatal intensive care unit (NICU), 1st and 5th minute Apgar scores, newborn gender, and UtA PI and UA PI Doppler results.

Exclusion criteria were as follows: chromosomal anomalies, genetic syndromes, major fetal anomalies, multiple pregnancies, fetal death, presence of maternal systemic diseases (chronic liver disease, SLE, diabetes mellitus, renal failure, autoimmune diseases, hypo-hyperthyroidism, cardiovascular diseases, and infections).

Blood samples for the study were collected from the case group after diagnosis of preeclampsia and admission to the hospital, prior to the initiation of any medical treatment such as magnesium sulfate or antenatal corticosteroids. For the control group, blood samples were obtained at the time of presentation to the outpatient clinic for routine check-ups.

To obtain the plasma phase, venous blood samples were taken and centrifuged at 1,000g for 15 min at 2-8°C and stored at −80°C until measurement. Quantitative measurements were made using the Mfn-2 ELISA kit. The detection sequence of the kits was 0.16 ng/mL-10 ng/mL and the sensitivity was 0.055 ng/mL.

### Statistical analysis

In this study, the primary outcome measure for patients with preeclampsia was Mfn-2 levels.

Data were analyzed using the SPSS 22.0 statistical program. The distribution of data was analyzed with the Shapiro-Wilk test. Minimum-maximum and median values were used for data that did not fit the normal distribution. Number and percentage values were given for categorical variables. Mann-Whitney U test was used in paired groups that did not show a normal distribution. ANOVA test was used for normally distributed data in comparison to more than three independent groups. The Kruskal-Wallis test was used for data that were not normally distributed. Tukey and Tamhane T2 tests were used for post-hoc analysis. Spearman's rank test was used in the correlation analysis of the data that did not fit the normal distribution. Categorical variables were appraised with the Pearson chi-square test. Receiver operating characteristic (ROC) analysis was performed to determine the threshold value of Mfn-2 levels in the prediction of preeclampsia. The effect of Mfn-2 level on the development of preeclampsia was assessed by binary logistic regression analysis. The limit of significance was taken as p≤0.05

## RESULTS

The laboratory and clinical results according to the groups are shown in Table 1. In the study, there was no difference between the groups in terms of age, gravida, parity, history of abortion, BMI, and gestational week (p>0.05). In terms of SBP and DBP values, all three groups were significantly different from each other. The lowest SBP-DBP values were in the healthy control group, and the highest values were in the severe group (p<0.05).

**Table 1:** Demographic and clinical characteristics of the patients grouped according to severity of preeclampsia.

	Groups				
	Total (n=90)	Control (n=30)	Mild PE (n=30)	Severe PE (n=30)	p
Age (years)†	26.4±6.3	25.0±4.1	26.4±5.3	27.7±8.5	0.171*
Gravida‡	3 (1-5)	3 (1-5)	3 (1-4)	2 (1-5)	0.235**
Parity‡	1 (0-4)	1 (0-4)	1 (0-3)	1 (0-4)	0.476**
Abortion story§	44 (36.4)	14 (35.0)	16 (38.1)	14 (35.9)	0.956***
Gestational week (weeks)‡	30.0±1.6	30.1±1.4	29.8±1.4	30.1±2.0	0.549*
BMI (kg/m^2^)‡	24.9±3.2	24.6±2.6	24.4±2.7	25.9±3.9	0.072*
SBP (mmHg)‡	146 (100-210)	120 (70-100)	146.5 (130-160)	170 (160-210)	0.000**
DBP (mmHg)‡	93 (70-120)	81.5 (70-100)	92 (80-105)	110 (100-120)	0.000**
UA PI‡	0.9 (0.5-1.7)	0.9 (0.6-1.2)	0.8 (0.5-1.7)	1.0 (0.7-1.4)	0.000**
Ut A PI‡	1.1 (0.6-1.1)	0.8 (0.6-1.8)	1.1 (0.7-1.7)	1.6 (0.7-2.1)	0.000**
AST (IU/l)‡	26 (12-120)	22 (12-45)	23 (13-43)	85 (44-120)	0.000**
ALT (IU/l)‡	32 (10-100)	21 (10-37)	23 (13-39)	78 (40-100)	0.000**
Platelets (IU/L)	211.6±69.8	251.3±52.5	242.9±54.1	137.1±31.6	0.000*
Proteinuria in 24 h (mg/dL)	752.1±354	0±0	1,101.9±02	1,132.9±632	0.000*
Creatinine (mg/dL)‡	0.5 (0.3-1.0)	0.5 (0.3-1.0)	0.5 (0.3-0.8)	0.7 (0.3-1.0)	0.000**
Mitofusin-2 (ng/mL)‡	2.22 (0.3-9.9)	1,11 (0.34-2.60)	2.22 (0.30-4.60)	5.1 (2.10-9.90)	0.000**

† mean±standard deviation

‡ median (min-max)

§ n (%). BMI: body mass index

4 SBP: systolic blood pressure

5 DBP: diastolic blood pressure

7 AST: aspartate aminotransferase

8 ALT: alanine aminotransferase

9 UtA: uterine artery

10 UA: umbilical artery

11 PI: pulsatility index

* One-way ANOVA

** Kruskal-Wallis test

*** Pearson chi-square, p-value <0.05 is significant

Notably, 24-h urine protein amounts increased from the control group to the sPE group. In terms of protein amounts in 24-h urine, the severe and mPE groups were similar but significantly higher than the control group (p<0.05).

UA PI and UtA PIs were similar in the control and mPE groups. It was significantly high in the sPE group than in the other groups (p<0.05).

There was a significant differentiation between the three groups in terms of AST and ALT levels. While the AST-ALT levels of the control and mPE groups were similar, the AST-ALT levels of the severe group were higher than the other two groups (p<0.05).

The control and mPE groups were similar in terms of platelet counts. Platelet counts of the severe group were lower than in the other groups (p<0.05).

In terms of creatinine levels, control 0.5 (0.3-1.0 mg/dL) and mPE 0.5 (0.3-0.8 mg/dL) groups were similar. However, the creatinine level in the sPE group 0.7 (0.3-1.0 mg/dL) was significantly higher than the other two groups (p<0.05).

Mfn-2 levels were different in all three groups. Mfn-2 levels rose from the control group to the sPE group (p<0.05).

Control and preeclampsia patient groups were statistically different in terms of the week of birth, birth weight, 1st and 5th minute Apgar scores, and NICU (p<0.001). The week of birth, birth weight, and Apgar values of the preeclampsia group were lower than those of the control group, and the NICU was higher (Table 2).

**Table 2:** Perinatal outcomes by groups.

	Groups		p-value
	Control (n=30)	Preeclampsia (n=60)	
Birth week (week)‡	39.0 (38.0-40.0)	34.0 (28.0-38.0)	0.000*
Birth weight (g)‡	3,400 (2,890:4,020)	2,500 (1,900-3,910)	0.000*
APGAR 1 min‡	8 (6:8)	8 (4:8)	0.001*
APGAR 5 min‡	10 (8:10)	9 (6:10)	0.001*
NICU§	5 (12.5)	27 (33.3)	0.015**

NICU: neonatal intensive care unit;

‡ Median (min-max);

§ n (%)

* Kruskal-Wallis test

** Pearson chi-square, p-value <0.05 is significant.

In the correlation analysis, there was no relationship between Mfn-2 levels and age, gravida, and BMI (p>0.001). Mfn-2 levels with SBP (r=0.696, p=0.000) and DBP (r=0.660, p=0.000), UA PI (r=0.238, p=0.008) and UtA PI (r=0.543, p=0.007) had a positive correlation (p<0.001). A relatively weaker association was found with UA compared to UtA PI. Mfn-2 values were negatively related to week of birth (r=-0.733, p=0.000) and birth weight (r=-0.637, p=0.000) and (p<0.001).

**Figure 1:**
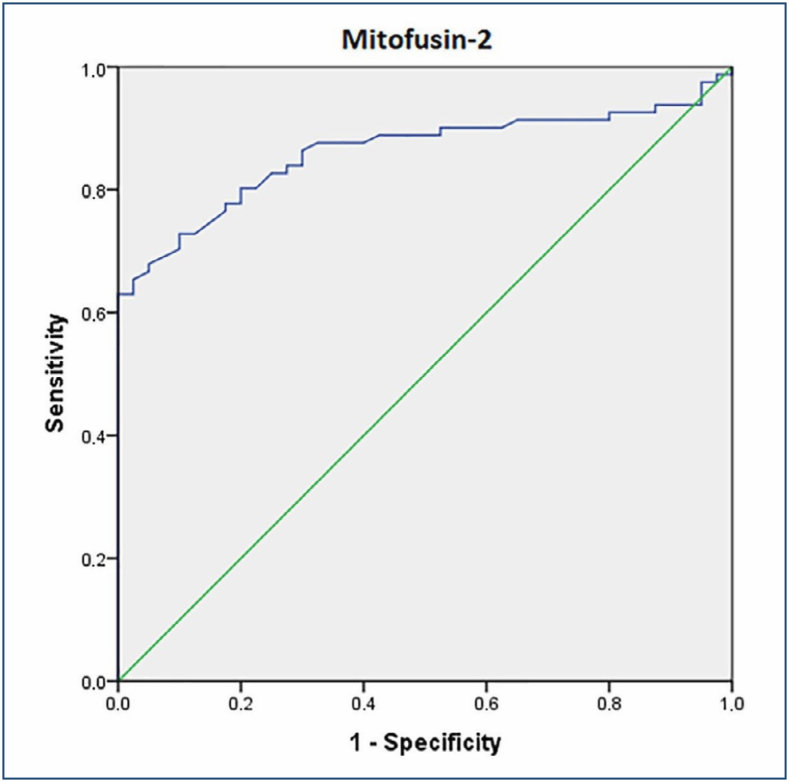
Receiver operating characteristic curve of mitofusin-2 in predicting preeclampsia: optimal cutoff 1.6 ng/mL; area under the curve: 0.861; 95%CI: 0.786-0.917; sensitivity: 83.9%; specificity: 70.0%; p≤0.001.

The effect of Mfn-2 in the diagnosis of preeclampsia was defined by the ROC curve (Figure 1). Mfn-2 values >1.6 ng/mL were significantly associated with an increased risk of sPE (p≤0.001).

A one-unit increase in Mfn-2 resulted in a statistically significant 4.21-fold increase in preeclampsia risk in logistic regression analysis.

## DISCUSSION

There is substantial evidence regarding the risk factors for preeclampsia; however, interpreting them is complex. Preeclampsia is a pregnancy-related heterogeneous disease whose etiology is not fully understood. How angiogenic imbalance occurs, leading to placental insufficiency, abnormal spiral artery remodeling, abnormal placentation, and what causes them are not known, and the molecular mechanism behind the development of preeclampsia remains unclear. There are numerous risk factors, including immunological factors, congenital anomalies, a family history of preeclampsia, chronic hypertension, kidney disease, diabetes, insulin resistance, genetic factors, nulliparity, high BMI, advanced maternal age, hydatidiform mole, and stress, among others. Screening for preeclampsia is challenging due to its asymptomatic nature. Reliable and accurate biomarkers will be necessary to predict the development of preeclampsia in the third trimester. Imaging tests such as uterine artery Doppler ultrasound allow the prediction of only one-third of preeclamptic cases. Since no single test is available to predict preeclampsia, a combination of tests is used to evaluate the condition. The most commonly used biomarkers for preeclampsia are sEng, sFlt-1, PlGF, and VEGF. It has been found that decreased PlGF levels in the first trimester along with increased sFlt-1 and sEng levels are associated with the development of preeclampsia. Studies suggest that the relationship between sFlt-1/PlGF is promising in indicating the imbalance between angiogenic and anti-angiogenic factors^9-11^.

In recent years, significant research has been conducted to illuminate the pathophysiology of the disorder, develop methods for identifying women at risk using predictive models, and investigate potential preventive strategies to reduce the incidence of preeclampsia. The ACOG and the International Society for the Study of Hypertension in Pregnancy (ISSHP) recommend screening pregnant women for PE in the first trimester. The primary goal of the first-trimester screening is to identify women at high risk of developing PE in the later stages of pregnancy, thereby enabling the implementation of appropriate preventive strategies. Currently, many centers do not use a unified first-trimester screening approach. Combining clinical risk factors, maternal blood pressure [mean arterial pressure (MAP), mean uterine arterial pressure (UtA), pulsatility index (PI)], and maternal angiogenic biomarkers (PlGF) in an algorithm may be a more accurate way to identify high-risk women^12,13^.

Despite all the studies, the molecules that contribute to the pathophysiology of preeclampsia have not been fully clarified. In this study, we tried to identify a new pathogenic factor that contributes to the development of PE pathogenesis. This is the first study to investigate maternal serum plasma Mfn-2 levels in patients with sPE.

Mfn-2 is a mitochondrial membrane protein that maintains mitochondrial bioenergy and promotes membrane fusion. Mfn-2 plays an important role in the regulation of cell proliferation and oxidative metabolism in many cell types^14^. In recent years, interest in studies related to Mfn-2 has increased, and the role of Mfn-2 in many mitochondria-related diseases has been mentioned^14^. The etiology and pathogenic mechanisms of PE are still unknown. Pathologies in mitochondrial mechanisms play an important role in the early stages of PE^5^.

Mitochondrial hypoxia, together with insufficient trophoblast invasion and unsuccessful spiral artery remodeling, is thought to cause the development of PE. Recent evidence suggests that placental mitochondrial problems may increase the development of PE. Oxidative stress caused by ROS that occurs due to mitochondrial dysfunction has been associated with PE. It has been reported that antioxidant protection decreased and ROS production increased in the placental mitochondria of women diagnosed with PE^15^.

Leboucher et al., in their studies on the role of Mfn-2 in the activation of mitochondrial apoptosis in cells, stated that mitochondrial dysfunction occurs due to placental hypoxia, and in this case, Mfn-2 levels decrease, which leads to mitochondrial fragmentation and apoptosis^16^.

Koziel et al., as a result of their study on the etiopathogenesis of PE patients, stated that placental hypoxia causes maternal endothelial dysfunction. In the study, it was also observed that there were adaptive changes in the mitochondria in the case of hypoxia in maternal endothelial cells^17^.

So far, studies on Mfn-2 and preeclampsia seem to be mostly of placental origin. This is the first study in the literature to examine serum maternal Mfn-2 levels in the patient group with sPE. In the study, significant differentiation was observed between sPE, mPE, and control patients in terms of Mfn-2 levels, and the highest values were observed first in the severe group and then in the mild group. Considering the perinatal outcomes between the preeclampsia and control groups, significant differentiation was observed in terms of the week of birth, birth weight, Apgar scores, and NICU. While there was a positive relationship between Mfn-2 levels and SBP and DBP, there was a negative relationship between birth week and birth weight. In this study, it was determined that a 1.6 ng/mL value of Mfn-2 could predict preeclampsia with 83.9% sensitivity and 70% specificity. A one-unit increase in Mfn-2 resulted in a statistically significant 4.21-fold increase in preeclampsia risk in logistic regression analysis.

Significant hemodynamic changes may occur during pregnancy, and the mother's hemodynamic profile is of great importance in treating hypertension in PE^18^. Maternal uterine arterial blood flow, one of the main factors in protecting the intrauterine environment, is necessary for maintaining fetal growth and development and normal placental function. However, hemodynamic ultrasound can predict pregnancy outcomes in PE patients and provide theoretical support to reduce adverse pregnancies. Color Doppler ultrasound can distinguish hemodynamic changes in the middle cerebral artery, UA, UtA, and middle and venous catheters. The PI of the UA and UtAPI are clinical markers of circulation to and from the placenta. Increased PI of the umbilical and uterine arteries and decreased PI of the fetal middle cerebral artery have been associated with fetal growth restriction due to placental insufficiency. Increased PI of the uterine arteries is also used to predict preeclampsia^19^.

In a study in which fetal Doppler ultrasound was used with a marker at the molecular level in severe preeclampsia patients, serum adiponectin and UA PI values were examined, and in the sPE group, increased UA PI was observed in parallel with the decreasing adiponectin levels, and a negative correlation was observed between both^20^. In our study, another specific one was observed. We detected increased UtA PI and UA PI in parallel with the serum Mfn-2 molecule. In the study, Mfn-2 levels, UA PI (r=0.238, p=0.008) and UtA PI (r=0.543, p=0.007) had a positive correlation (p<0.001). A relatively weaker association was found with UA compared to UtA PI.

In PE, vascular invasion is inadequate, leading to reduced placental perfusion, ultimately resulting in chronic hypoxia and intrauterine growth restriction. Oxygen deficiency results in the production of anti-angiogenic factors such as sFlt-1, sEng, TGF-β1, and TGF-β3 by the placenta. These factors enter the maternal circulation, resulting in endothelial dysfunction, hypertension, and proteinuria^21^.

Mitochondria, known as the powerhouse of the cell, are vital organelles for energy production and maintaining cellular dynamics. Mitochondria consume the most oxygen in cells to produce ATP through oxidative phosphorylation. Hypoxia affects mitochondrial function because it disrupts ATP production and increases mitochondrial ROS (mROS). Since mitofusin-2 (Mfn-2) is a mitochondrial fusion protein located on the outer membrane of the organelle, changes in both Mfn-2 and ATP expression in the placenta of preeclamptic patients indicate the relationship between hypoxia and mitochondrial dysfunction^17^.

In our study, we also found that levels of Mfn-2 increased in preeclampsia patients parallel to increased anti-angiogenic factors such as sFlt-1 and sEng, indicating increased endothelial dysfunction due to hypoxia.

When looking at the literature, it has been found that the biomarker HIF-1α, which is associated with placental hypoxia and has been the subject of recent studies, plays a crucial role in the pathogenesis of PE. HIF-1α achieves this by facilitating anti-angiogenic activation (VEGF, PlGF) and inhibiting pro-angiogenic factors (sFlt-1, sEng). Additionally, HIF-1α increases the expression of p38MAPK and NLRP3 inflammasomes, which promote placental inflammation and dysfunction^22^. Similar to HIF-1α, Mfn-2 also shows an increase secondary to placental hypoxia, playing a role in the pathophysiology of preeclampsia as a key transcription factor regulating cellular responses to hypoxia and low oxygen tension.

Micronutrients (iron, calcium) and antioxidant deficiencies (vitamins C and E) may contribute to the development of preeclampsia/eclampsia. Iron (Fe) and calcium (Ca) deficiencies have been reported to increase the risk of preeclampsia in women. Studies have suggested that pregnant women with low calcium levels may benefit from daily supplementation of 1.5-2 g of calcium, as it has been shown to reduce the incidence of preeclampsia, maternal death, and serious morbidity. On the contrary, supplementation of antioxidants such as omega-3 vitamins C and E, or selenium does not seem to prevent preeclampsia and is currently not supported by evidence^23^.

Aspirin is currently the only medication recommended for the prevention of preeclampsia. Both the US Preventive Services Task Force (USPSTF) and the ACOG recommend the use of aspirin for the prevention of preeclampsia in women at high risk of developing the condition (such as those with chronic hypertension, pregestational diabetes, multiple gestations, kidney disease, and autoimmune disease) between weeks 12 and 28 of pregnancy^24^.

The expected management of preeclampsia focuses on reducing the risk of maternal and neonatal complications through the administration of antihypertensive agents (either as a single agent or as a combination of two, such as nifedipine/methyldopa/labetalol/hydralazine) and anticonvulsants (such as magnesium sulfate). Antihypertensive medications reduce maternal complications such as cerebral hemorrhage, eclampsia, and acute pulmonary edema, while anticonvulsants reduce complications of eclampsia for both the mother and the newborn. Depending on the gestational age, the use of betamethasone (two injections of 12 mg each, 24 h apart) aids in fetal lung maturation and reduces the risk of neonatal complications such as hyaline membrane disease, intraventricular hemorrhage, and neonatal mortality. The treatment of preeclampsia depends on the stage of pregnancy. If severe preeclampsia is detected before the 24th week of pregnancy, termination of the pregnancy is recommended due to the high risk of maternal complications and poor neonatal prognosis. Management between weeks 24-34 and 34-37 of gestation depends on the severity of preeclampsia. Expectant management is recommended for mPE, but in the presence of severe preeclampsia or uncontrolled severe hypertension (not responding to dual therapy), symptoms such as acute pulmonary edema, subcapsular hepatic hematoma, placental abruption, or eclampsia, the most effective treatment method is urgent delivery^25^.

It is evident that the current study has some potential limitations, and the resulting findings will contribute to future research. Examples of limitations in the study include being a single-center study and having a relatively small sample size. Additionally, in this study, blood samples were collected only at the time of the initial diagnosis of preeclampsia. Therefore, other factors limiting our study include not evaluating the serum Mfn-2 concentrations before the onset of preeclampsia and not monitoring changes in serum concentrations until delivery. A study design could be planned where blood samples are collected at the time of diagnosis and during follow-up. Subsequently, multiple plasma samples could be obtained to determine if there are changes in Mfn-2 levels over time.

In conclusion, we found that serum Mfn-2 concentrations, an important mitochondrial biomarker, are significantly elevated in women with pregnancy complicated by preeclampsia compared to healthy controls, with this elevation being more pronounced in severe preeclampsia. Our study provides a new perspective on the role of Mfn-2 as a novel biomarker for maternal and fetal/neonatal complications in women with suspected or confirmed preeclampsia. It is possible that full elucidation of the pathophysiology may not be achievable with the factor studied. However, this biomarker we investigated is a specific indicator, and further studies are needed to clarify the pathophysiology.

## CONCLUSION

The severity of the disease increases due to increased placental hypoxia and endothelial dysfunction in patients with preeclampsia, and it is known that the patient has more advanced pathological results. In this study, we detected more elevated serum maternal Mfn-2 levels in response to increased hypoxia and endothelial dysfunction in sPE. Current management of PE is based on the diagnosis and assessment of the disease and the appropriate timing of delivery. We think that the use of diagnostic tools such as ultrasonographic Doppler findings together with serum plasma Mfn-2 may be promising in predicting diagnosis and treatment. We think that this study is important as it can be a source for studies to be conducted with larger patient groups and to reveal this important relationship between sPE and Mfn-2.
